# A Systematic Review of the Use of Janus Kinase Inhibitors in Large Vessel Vasculitis

**DOI:** 10.7759/cureus.91235

**Published:** 2025-08-29

**Authors:** Timmie Chay, Tirath Patel, Nabina Dumaru, Srivarshini Maddukuri, Ryan R Haddad, Naga Spandana Battula, Lubna Mohammed

**Affiliations:** 1 Medicine, Tseung Kwan O Hospital, Hong Kong, HKG; 2 Medicine, American University of Antigua, Saint John's, ATG; 3 Radiology, Frimley Park Hospital, Frimley, GBR; 4 Internal Medicine, Dr. D. Y. Patil Medical College, Hospital and Research Centre, Pune, IND; 5 General Practice, Jordan Medical Council, Amman, JOR; 6 Clinical Research, California Institute of Behavioral Neurosciences & Psychology, Fairfield, USA; 7 Internal Medicine, Dr. VRK Women's Medical College, Hyderabad, IND

**Keywords:** baricitinib, biologics therapy, giant cell arteritis (gca), janus kinase inhibitors, large vessel vasculitis, refractory disease, ruxolitinib, takayasu arteritis (tak), tofacitinib, upadacitinib

## Abstract

Janus kinase inhibitors (JAKi) are emerging agents in the treatment of rheumatological diseases. Their role in rheumatoid arthritis and spondyloarthropathies has been established. The treatment of Takayasu Arteritis (TAK) and Giant Cell Arteritis (GCA) is large vessel vasculitis requiring long courses of glucocorticoids, often combined with other immunosuppressants. It has been shown that the pathogenesis of TAK and GCA involves the JAK-signal transducer and activator of transcription (STAT) pathway, showing that JAKi can theoretically be efficacious in these conditions. A systematic review of the use of JAKi in TAK and GCA is conducted across six databases: PubMed, PubMed Central, Google Scholar, EMBASE (via OVID), Science Direct, and Cochrane Library. Studies in the English language and human studies from 2019 to 2024 on the efficacy and safety of JAKi in patients with GCA or TAK were included. Pre-clinical, in-vitro, animal studies, or studies in which patients have other concomitant autoimmune diseases, malignancies, or prior exposure to JAKi are excluded. In total, 19 studies were included, of which six were cohort studies and seven were case reports for TAK. For GCA, three cohort studies, two case reports and one double-blind randomized controlled trial (RCT) were identified. One case report involves an overlap between TAK and GCA, but apart from older age, the patient only had TAK features. Abstracts and patients with other concomitant autoimmune diseases or malignancies were excluded. Upadacitinib, tofacitinib, baricitinib, and ruxolitinib were the JAKi studied. Of the cohort studies, two included a comparison group with traditional immunosuppressants, namely, methotrexate and leflunomide, and both were in TAK. All case reports were about patients with refractory disease. The only RCT was done with GCA patients using upadacitinib 15mg daily vs 7.5mg daily vs placebo. Most studies and case reports demonstrated the effectiveness of JAKi in the outcomes measured, namely, clinical activity, symptoms or signs, imaging findings, inflammatory marker levels, disease relapse, and corticosteroid requirement. JAKi are also generally safe, with infections being the most common adverse effect. This review is limited by the fact that most studies do not have a controlled group and that the different definitions of disease remission, relapse, or refractoriness are adopted across different studies. Thus, future studies addressing these limitations are needed.

## Introduction and background

Takayasu arteritis (TAK) and giant cell arteritis (GCA) are autoimmune diseases characterized by granulomatous inflammation of large vessels, namely, the aorta and its major branches. While both diseases are more common among females and may present with pyrexia of unknown origin, weight loss, and malaise [1], GCA usually affects older individuals (>50 years old), while patients with TAK are more often under 40 years old [2].

GCA is diagnosed with clinical symptoms and signs, including new temporal headache, scalp tenderness, sudden visual loss, jaw or tongue claudication, abnormal examination of the temporal artery, and stiffness in the neck and shoulder. Elevated inflammatory markers, positive imaging findings, and temporal artery biopsy results are also important for diagnosis [3]. TAK usually presents with angina, arm or leg claudication, vascular bruit, diminished upper extremity pulses, carotid artery abnormalities, and a difference in systolic blood pressure between the arms. Evidence of vasculitis on imaging is needed to confirm the diagnosis of TAK, while raised inflammatory markers support its diagnosis [4]. Without prompt treatment, complications such as blindness, stroke, myocardial infarction, heart failure, aortic regurgitation, aneurysm formation, and even rupture may occur [5,6].

For the treatment of GCA, current guidelines suggest that high-dose glucocorticoids be started together with tocilizumab, followed by gradual tapering of steroids. If the disease is refractory to steroids, other immunosuppressants such as tocilizumab (if not already added), methotrexate, or abatacept can be considered. As for TAK, high-dose steroids and non-glucocorticoid immunosuppressants, such as methotrexate, azathioprine, tumor necrosis factor (TNF) inhibitors, and tocilizumab, are recommended as the first-line treatment. If the disease is not well controlled or relapses, escalation of immunosuppression is recommended [7].

However, studies have shown that glucocorticoids alone can achieve disease remission in only 25-50% of cases of TAK [8]. Up to 93% of patients may relapse in the first two years, especially during the reduction of steroids [2]. Even with the addition of immunosuppressants as induction treatment, around 54% of patients may have refractory disease [9].

In patients with GCA, more than 40% of patients may suffer from a relapse, particularly in the first two years [10]. As mentioned above, tocilizumab is one of the first-line agents in induction therapy recommended in the latest guideline, but studies have shown that around one-third to half of the patients do not achieve admission after 12 months of therapy [11]. Moreover, around 10%-38% of patients may relapse while still receiving tocilizumab, and around one-third to two-thirds of patients who stop tocilizumab may have relapsing disease [10]. It is therefore important and necessary to explore other potential agents that may control GCA and TAK, particularly in relapsing or refractory disease.

To do so, we must return to the pathogenesis of TAK and GCA, which involves many cytokines and interferons binding to various receptors and activating intracellular pathways. One of the most important pathways is the Janus kinase inhibitor (JAKi)-signal transducer and activator of transcription(JAK-STAT) pathway. Cytokines and interferons bind to receptors of the JAK-signal transducer on the cell surface and activate the JAK-STAT pathway. The transcription factor, STAT, is subsequently transported to the nucleus, where it regulates gene expression [12], ultimately leading to inflammation of the intima, media, and adventitia and formation of granulomatous lesions. Remodeling processes eventually lead to the formation of aneurysms or intimal hyperplasia, causing lumen occlusion [13].

As evidenced by the effectiveness of tocilizumab (an anti-IL6 agent), IL-6 plays a paramount role. IL-6 binds to JAK1, JAK2, and TYK2 receptors, which then activate STAT3 pathways [12]. This promotes the development of Th17, which in turn produces a number of cytokines that activate systemic inflammatory responses. Some of these cytokines further stimulate dendritic cells and macrophages to produce more IL-12, which induces T cells to differentiate into Th1 cells, of which the signature cytokine is IFN-γ. IFN-γ employs JAK1 and JAK2 and ultimately contributes to vascular remodelling, intimal hyperplasia, and luminal occlusion - a phenomenon more commonly seen in TAK. This may therefore explain the higher levels of IFN-γ found in TAK than in GCA. Higher levels of IFN-γ are also found in subtypes of GCA with vascular occlusion, such as cranial GCA with ischemic event and large vessel GCA, which shares common features with TAK [14].

Furthermore, levels of type I interferons have been shown to be elevated in GCA [15] and TAK [16]. Type 1 interferons activate JAK1, JAK2, and TYK2 receptors, which then trigger STAT1 and STAT2 activation [13,17]. Other cytokines, such as GM-CSF in GCA and those of TNF-α, IL-2, IL-3, and IL-4 in TAK, which are all ligands to JAK1, JAK2, or JAK3, have also been reported [17-19].

It can be seen that JAKi block many downstream signals from different cytokines and interferons, predicting their usefulness in TAK and GCA, particularly in refractory or relapsing disease.

Murine GCA models have shown the effectiveness of tofacitinib in suppressing T cell proliferation, adventitial angiogenesis, and intimal hyperplasia [20]. Ruxolitinib and tofacitinib have been shown in in vitro experiments to be effective in reducing T cell proliferation and STAT signals in peripheral blood mononuclear cells from patients with TAK [16].

So far, tofacitinib, baricitinib, and upadacitinib are the JAKi most commonly used in rheumatological diseases [21]. Since ruxolitinib has been shown in in vitro studies to be effective in patients with TA, it will also be evaluated here. Tofacitinib mainly targets JAK1 and JAK3. Upadacitinib selectively targets JAK1, while baricitinib and ruxolitinib target JAK1 and JAK2 [22]. Based on current knowledge in the pathogenesis of GCA and TAK, as well as in vitro study evidence, JAKi are potential agents for the treatment of GCA and TAK. This study therefore aims to study the effectiveness of JAKi in the refractory or relapsing GCA and TAK.

## Review

Methods

This systematic review aims to evaluate the efficacy and safety of JAKi, namely, tofacitinib, baricitinib, upadacitinib, and ruxolitinib, in the treatment of refractory or relapsing GCA and TAK. This review follows the Preferred Reporting Items for Systematic Reviews and Meta-Analyses (PRISMA) 2020 recommendations. 

Inclusion Criteria

Studies in the English language and human studies from 2019 to 2024 were selected for the review. Studies concerning the evidence, use, efficacy, and safety of JAKi in patients with GCA or TAK were included. Patients are diagnosed to have GCA with ACR 1990 criteria, 2016 Revised ACR criteria (rACR) for diagnosis of GCA, 2022 ACR/EULAR GCA classification criteria, Chapel Hill Consensus Conference definition for GCA, or have a clinical diagnosis of GCA. Patients with TAK are diagnosed with ACR 1990 criteria, 2022 ACR/EULAR Takayasu’s arteritis classification criteria, Ishikawa’s criteria, Sharma’s modification of Ishikawa’s criteria, and Chapel Hill Consensus Conference definition for TAK or are clinically diagnosed to have TAK.

Types of the Study Included

Randomized controlled trials, case series, observational studies, and case reports with outcomes measured with inflammatory markers, disease activity, or imaging studies are included.

*Exclusion Criteria* 

Pre-clinical/in-vitro/animal studies of JAKi on vasculitis are excluded. Studies in which patients have other concomitant autoimmune diseases, malignancies, or prior exposure to JAKi are also excluded.

Literature Search

Literature searches were done on PubMed, PubMed Central (PMC), EMBASE (via OVID), Google Scholar, Science Direct, and Cochrane Review (search strategy in Appendix, Table 1). Keywords used were “Large vessel vasculitis”, “Takayasu’s arteritis”, or “Takayasu arteritis”, “Giant cell arteritis”, “Janus kinase inhibitors” or “JAK inhibitors”, “Upadacitinib”, “Baricitinib”, “Tofacitinib”, “Ruxolitinib”, “Effectiveness”, “Efficacy”, “Evidence”, “Safety”, and “Use”. MeSH terms and a combination of keywords were used in searches. Results were downloaded to EndNote Reference Manager, and duplicates were removed. After certain articles were eliminated based on title and abstract, the remaining ones were screened after full texts were reviewed.

Outcome Analysis 

Outcomes are analyzed in terms of clinical improvement, ESR and CRP levels, rate of relapse, imaging studies (MRA, CTA, US, PET-CT), glucocorticoid dosage, and presence of adverse events. Clinical improvement is defined as improvement or remission in clinical symptoms and signs, or improvement in disease activity scores. Refractory disease is defined as “persistent active disease despite an appropriate course of immunosuppressive therapy,” and relapse is defined as “recurrence of active disease following a period of remission”, according to the 2021 ACR guideline [7].

Quality of the Included Studies

The quality of cohort studies was assessed by the Newcastle-Ottawa Scale (NOS). There are nine cohort studies in total. Only two studies had a comparison group, so only these two could be scored under “selection of non-exposed group” and comparability criteria. All the studies scored in other items and criteria under the NOS. Details can be referred to Table 1.

**Table 1:** Quality of cohort studies *: criterion fulfilled; 0: criterion not fulfilled

Studies	Type	Selection 1, 2, 3, 4	Comparability 1, 2	Outcome 1, 2, 3
Dai et al. [23]	Prospective	* 0 * *	0 0	* * *
Kong et al. [24]	Prospective	* * * *	* (age sex) 0	* * *
Mv et al. [25]	Prospective	* 0 * *	0 0	* * *
Wang et al. [26]	Prospective	* * * *	* * (sex age) (disease activity at baseline)	* * *
Zhou et al. [27]	Prospective	* 0 * *	0 0	* * *
Li et al. [28]	Prospective	* 0 * *	0 0	* * *
Koster et al. [11]	Prospective	* 0 * *	0 0	* * *
Eriksson et al. [29]	Retrospective	* 0 * *	0 0	* * *
Loricera et al. [30]	Retrospective	* 0 * *	0 0	* * *

Case reports are evaluated by the Joanna Briggs Institute Critical Appraisal Checklist for case reports (JBI). There are nine case reports in total. Two scored seven out of eight, as they did not have information on whether any adverse effects were experienced. Other reports scored full. Two scored six out of eight as they did not have detailed information about the patients’ history and adverse effects. Details of the quality of case reports can be referred to Table 2.

**Table 2:** Quality of case reports JBI: Joanna Briggs Institute Critical Appraisal Checklist for case reports; Q: question *: criterion fulfilled; 0: criterion not fulfilled

Study	Components of JBI								
	Q1	Q2	Q3	Q4	Q5	Q6	Q7	Q8	Total
Yang et al. [31]	*	*	*	*	*	*	*	*	8
Palermo et al. [32]	*	*	*	*	*	*	0	*	7
Yamamura et al. [33]	*	*	*	*	*	*	*	*	8
Wang et al. [34]	*	*	*	*	*	*	*	*	8
Régnier et al. [16]	*	0	*	*	*	*	0	*	6
Régent et al. [35]	*	*	*	*	*	*	*	*	8
Belfeki et al. [36]	*	*	*	*	*	*	*	*	8
Camellino et al. [37]	*	*	*	*	*	*	0	*	7
Prigent et al. [38]	*	0	*	*	*	*	0	*	6

The randomized controlled trial by Blockmans et al. [39] is appraised using the JBI Critical Appraisal Tool for Assessment of Risk of Bias for Randomized Controlled Trials. Results are in Table 3. The study scored in all items, except for Question 6, since patients randomized to upadacitinib groups were put on a 26-week steroid tapering schedule, while those on placebo were put on a 52-week steroid tapering schedule.

**Table 3:** Quality of RCT JBI: Joanna Briggs Institute Critical appraisal tool for assessment of risk fo bias for randomized controlled trials (RCTs); Y: Yes; N: No

	Components of JBI												
Question	1	2	3	4	5	6	7	8	9	10	11	12	13
	Y	Y	Y	Y	Y	N	Y	Y	Y	Y	Y	Y	Y

Results

**Figure 1:**
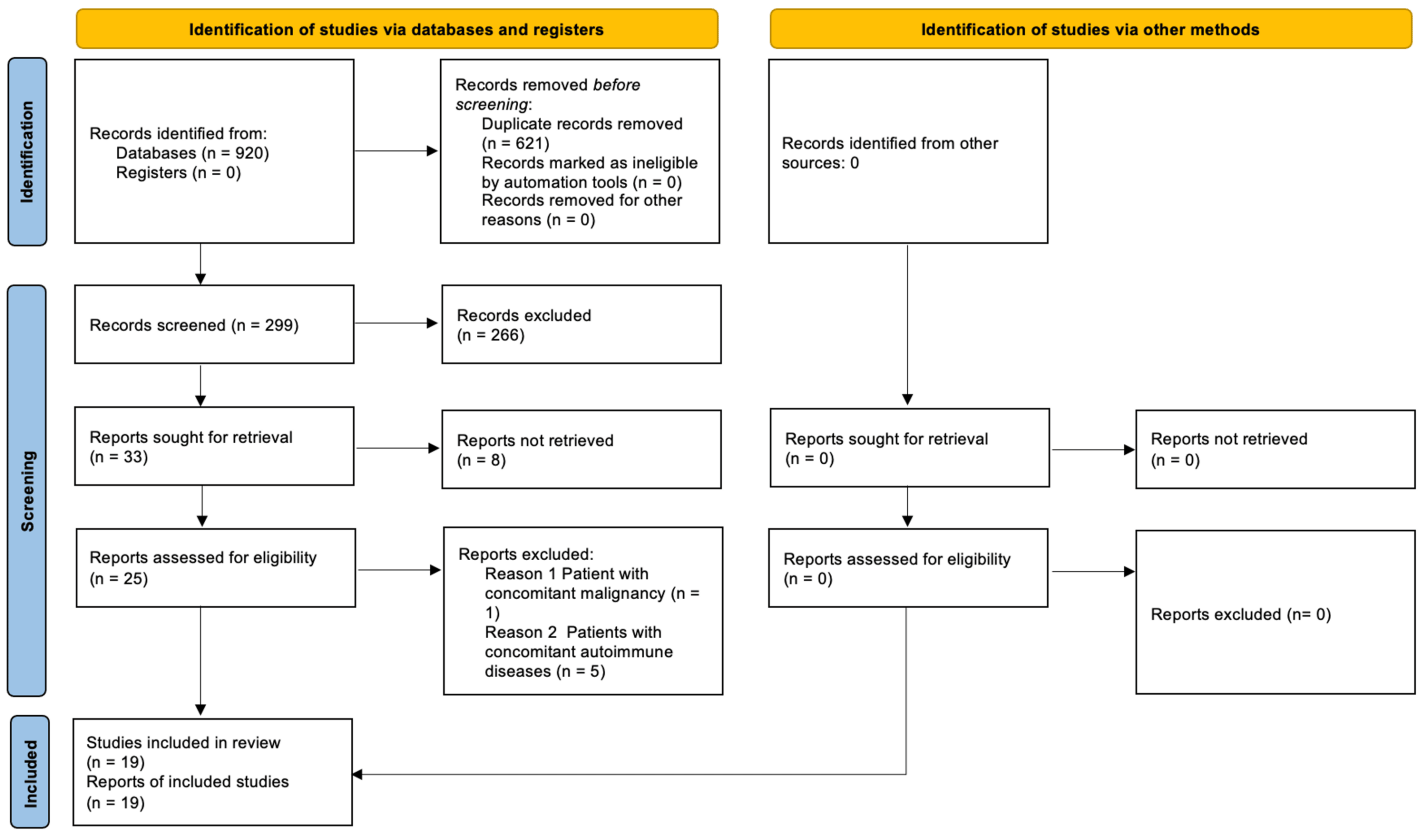
PRSIMA flow chart PRISMA: Preferred Reporting Items for Systematic Reviews and Meta-Analyses

A total of 920 records are identified from the databases, with 621 duplicated records. The remaining 299 records were screened based on titles and abstracts, and 266 were excluded. Full reports for the remaining 33 records were sought, but four were only scientific abstracts, and four were future studies. The 25 papers were studied for eligibility, from which six were excluded (Table 4). In one report [40], the patient had concomitant chronic neutrophilic leukemia. In the other six reports [41-45], patients had concomitant autoimmune diseases, namely, ulcerative colitis and psoriatic arthropathy. The PRISMA flowchart can be seen in Figure 1.

**Table 4:** Studies excluded TAK: Takayasu's arteritis; GCA: giant cell arteritis; JAKi: Janus kinase inhibitors

Study	TAK/GCA	JAKi studied	Concomitant condition
Herlihy et al. [40]	GCA	Ruxolitinib	Chronic neutrophilic leukemia
Ino et al. [41]	TAK	Tofacitinib	Ulcerative colitis
Kuwabara et al. [42]	TAK	Tofacitinib	Ulcerative colitis
Rios Rodriguez et al. [43]	TAK	Tofacitinib	Ulcerative colitis
Sato et al. [44]	TAK	Tofacitinib	Ulcerative colitis
Sanada et al. [45]	GCA	Upadacitinib	Psoriatic arthropathy

Study Characteristics

Takayasu arteritis: There are six prospective studies [23-28] and seven case reports [16,31-36] on TAK, with a total of 111 patients treated with JAKi. Most of the patients are treated with tofacitinib, followed by baricitinib, ruxolitinib, and upadacitinib. Régent et al. [35] reported a case of overlap between TAK and GCA. Since the patient had no GCA symptoms or signs, but had asymmetrical blood pressures, vascular bruits, aorta and subclavian uptake on PET-CT, she will be included in the discussion of TAK. Three studies include treatment-naive and treatment-refractory patients [23,24,26], but only two [24,26] showed separate analysis for treatment-naive and treatment-refractory patients. All other studies and reports are in patients with refractory disease. Two studies [24,26] compared JAKi (tofacitinib) with another immunosuppressant, namely, methotrexate and leflunomide, respectively. All studies reported outcomes in terms of clinical symptoms or signs or remission, as well as ESR and/or CRP levels. Five studies reported disease activity scores in terms of the National Institutes of Health (NIH) [16,23,24,26,31] and one in Indian Takayasu Clinical Activity Score (ITAS) [46], 11 studies [23,24,26-28,31-36] reported changes in imaging studies (ultrasound/MRA/CTA/PET-CT), and 10 studies [16,24-28,31,34-36] reported changes in glucocorticoid dosage. Detailed study characteristics are shown in Table 5.

**Table 5:** Takayasu arteritis study characteristics * Statistically significant JAKi: JAK inhibitors; GC: glucocorticoids; MTX: methotrexate; CYP: cyclophosphamide; MMF: mycophenolate mofetil; TCZ: tocilizumab; LEF: leflunomide; HCQ: hydroxychloroquine; AZA: azathioprine; INF: infliximab; TNFi: Tumor necrosis factor inhibitor; CYA: cyclosporine; RIT: rituximab; ADA: adalimumab; TOF: tofacitinib; BAR: baricitinib; RUX: ruxolitinib; UPA: upadacitinib; ESR: erythrocyte sedimentary rate; CRP: C-reactive protein; F: female; M: male; CTA: computer tomographic angiography; MRA: magnetic resonance angiography; PET-CT positron emission tomography computer tomography; US: ultrasound; LCCA: left common carotid artery; RCCA: right common carotid artery; LSCA: left subclavian artery; NIH: National Institute of Health; ITAS: Indian Takayasu Activity Score; TN: treatment naive; TR: treatment resistant; NA: not applicable; NS: not specified; SD: standard deviation; dx: diagnosis; P: prospective; R: retrospective

Study	Type	JAKi studied	Mean Age in years (SD)	No of patients (Gender)	Median duration of disease in months (IQR unless specified)	Duration of follow-up (P)/JAKi use (R)	Concomitant medications (apart from steroids)	TN:TR	Previous therapies	Outcomes					
Dai et al. [23]	Perspective	TOF	28.12±8.99	17 (14F, 3M)	15 (12-35)	12 months	No	6:11	GC alone 3 GC + MTX 1 GC + LEF 4 GC +MMF 2 GC + TCZ 1	Clinical NIH score ESR CRP, cytokines/chemokines imaging (MRA)					
Kong et al. [24] (TN vs TR group analyzed separately below)	Prospective	TOF	TOF: 31.11±9.58, MTX: 33.50±14.89	27 (GC + TOF), 22F 5M, 26 (GC + MTX), 23F 3M	TN: TOF 10.5 (2, 37.25); MTX 2.0 (1.00, 12.00). TR: TOF: 31.0 (15.0, 79.0); MTX: 36.0 (11.0, 60.0)	12 months	No	TOF group 8:19 vs MTX group 19:7 #	(TOF/MTX) GC: 6/0, GC+LEF: 5/2, GC+CYP:2/4, GC+MTX: 2/0, GC+MMF:1/1 GC+IL-6: 3/0	Clinical NIH score, ESR CRP imaging (MRA/CTA) GC dose					
Kong et al. [24] - TN Group	Prospective	TOF	TOF: 33.00±9.58, MTX: 34.21±15.65	TOF: 5F, 3M; MTX: 17F, 2M	TOF: 10.5 (2, 37.25), MTX: 2 (1, 12)	NA	NA	NA	NA	NA					
Kong et al. [24] - TR Group	Prospective	TOF	TOF: 30.32±9.73, MTX: 34.71±14.81	TOF: 17F, 2M; MTX: 6F, 1M	TOF: 31 (15, 79); MTX: 36 (11, 60)	NA	NA	NA	NA	NA					
Pakashini et al. [25]	Prospective	TOF	Mean: 28.3 (+- 9.35)	10 (9F, 1M)	Mean: 25.2±9.89 (SD)	6 months	NS	NA	MTX 1, MTX, MMF 6 AZA, MTX, MMF 2, MMF, Etanercept 1	Clinical ITAS score, ESR CRP GC					
Wang et al. [26] (TN vs TR group analyzed separately below)	Prospective	TOF	Total: 32.33 ±10.67, LEF: 33.23±12.07, TOF: 30.94±9.03	35 GC + LEF), 32 (GC + TOF)	Total: 14 (3, 59); LEF: 5 (1, 24); TOF: 30 (11, 68.75) #	12 months	None	LEF: 21:14; TOF: 9:23 #	NS	Clinical NIH E SR CRP MRA GC dose					
Wang et al. [26] - TN group	Prospective	TOF	LEF: 31.76±13.3, TOF: 33.56±9.11	LEF: 17F, 4M; TOF: 5F, 4M	LEF: 1 (1, 4.5); TOF: 15 (3, 36.5) *	NA	NA	NA	NA	NA					
Wang et al. [26] - TR group	Prospective	TOF	LEF: 35.43± 10.01, TOF: 30.48±9.08	LEF: 13F, 1M; TOF: 21F, 2M	LEF: 42 (10, 126); TOF: 31 (12, 74)	NA	NA	NA	NA	NA					
Zhou et al. [27]	Prospective	BAR	Median: 28 (22–37 IQR)	10 (9F, 1M)	Median duration of 50 (24–65)	Mean: 15.3 (range: 4–31) months	MTX 5 LEF 2 HCQ 1 MTX+HCQ 2	NA	MTX, LEF, MMF, HCQ, TAC, Sirolimus, TCZ, CYC, Secukinumab, TNFi, Tofacitinib	Clinical CRP ESR imaging (CTA, thickness of carotid and SCA by Doppler) GC					
Li et al. [28]	Prospective	TOF	22±4.58	5, all F	23 (11, 58.5)	6 (5 pt) - 18 months (1 pt)	NS	NNA	MTX 4, CYC 2, AZA 2, LEF, MMF 4, tacrolimus 1, tocilizumab 4	Clinical ESR CRP GC dose, vascular Doppler (artery stenosis and mural thickness)					
Yang et al. [31]	Case report	TOF	28	1F	10 years	33 months	MMF+TCZ (6 months), LEF (2 months)	NA	CYC MTX CYA LEF HCQ MMF TCZ	Clinical NIH ESR, CRP US, and contrast-enhanced US of LCCA/RCCA/LSCA thickness GC dose					
Palermo et al. [32]	Case report	TOF	23, 25	2 Both F	64 months, 11 yrs	2 & 7 months since TOF	2nd case: MMF	NA	1st: MTX, RIT, ADA, TCZ 2nd: MTX, INF, TCZ, AZA, MMF, ADA, RIT	Clinical ESR CRP, MRA, PET CT					
Yamamura et al. [33]	Case report	TOF	26	M	2 years	12 months	MTX	NA	INF, CYA, AZA, TCZ, CYC	Clinical ESR CRP MRA, PETCT					
Wang et al. [34]	Case report	TOF (extended release)	21	M	3 years	9 months	NS	NA	MTX, AZA	Clinical CRP, IL6, IL18, TNFa CT thickness of CCA, aorta					
Régnier et al. [16]	Case report	BAR, RUX	No current age	2F, 1M (has primary polycythemia)	(Age at dx: 50, 40, 30)	6 months	NS	1:2	MMF, TCZ, TNFi, MTX/ MMF, TCZ, TNFi	Clinical CRP ESR CTA GC dose					
Regent et al. [35]	Case report	BAR	52	1F	6 years	6 months	NS	NA	MTX, TCZ, INF, CYP	NIH score CRP GC dose CD 25, Th1/17, pSTAT levels					
Belfeki et al. [36]	Case report	UPA	33	1F	3 years	12 months	MTX, INF	NA	MTX, TCZ, INF	Clinical ESR CRP, PET-CT GC dose					

Giant cell arteritis (GCA)*: *Three cohort studies, of which one is prospective [11], two are retrospective [29,30], two case reports [37,39], and one double-blind randomized controlled trial are included. A case report [45] regarding the use of upadacitinib was excluded since the patient had concomitant psoriatic arthropathy. In total, 72 patients were studied in observational studies and case reports, with most patients treated with baricitinib, followed by tofacitinib and then upadacitinib. The case series by Camellino et al. [37] includes two patients with only polymyalgia rheumatica (PMR) with no vasculitis, two patients with PMR and GCA, and two patients with GCA. The studies and reports are on patients with refractory disease, meaning patients who do not respond to initial corticosteroid therapy, except for one patient in the report by Camellino et al. [37]. All studies reported outcomes in terms of clinical improvement and ESR/CRP levels. One study [11] included disease activity score using the Birmingham vasculitis activity score [47] and patient global assessment scale. Four [11,29,30,37] reported changes in glucocorticoid dosage, and one [38] reported changes in imaging (PET-CT). In the double-blind RCT by Blockmans et al. [39], 209 patients received upadacitinib 15 mg daily, 107 received upadacitinib 7.5 mg daily, and 112 received a placebo. The primary endpoint was sustained remission at week 52 (absence of signs or symptoms of GCA from week 12 to week 52 and adherence to the protocol-specified glucocorticoid taper. Secondary endpoints included sustained complete remission (CR) (sustained remission with normalization of ESR and CRP levels, flare-ups, glucocorticoid dosage, glucocorticoid-related adverse events, and patient-reported outcomes. Detailed study characteristics are listed in Table 6. 

**Table 6:** Giant cell arteritis study characteristics GC: glucocorticoids; MTX: methotrexate; CYP: cyclophosphamide; MMF: mycophenolate; TCZ: tocilizumab; LEF: leflunomide; HCQ: hydroxychloroquine; SFZ: sulfasalazine; TOF: tofacitinib; BAR: baricitinib; UPA: upadacitinib; ESR: erythrocyte sedimentary rate; CRP: C-reactive protein; UPA 7.5: upadacitinib 7.5 mg daily; UPA 15: upadacitinib 15 mg daily; F: female; M: male; (P): in prospective studies; (R): in retrospective studies; BVAS: Birmingham vasculitis activity score; PGA: patient global assessment; TN: treatment-naive; TR: rreatment resistant (GC with or without non GC immunosuppressant); NA: not applicable; NS: not specified: Pt: patient; PMR: polymyalgica rheumatica; GCA: giant cell arteritis *Eight in the baricitinib group, nine in the tofacitinib group, and nine in the upadacitinib group; †2 in the baricitinib group, 0 in the tofacitinib group, and one in the upadacitinib group ‡Pt 1 and 6 had PMR without vasculitis; Pt 2 and 5 had PMR and GCA; Pt 3 and 4 and GCA.

Study	Type	JAKi studied	Mean age in years (SD)	No. of patients (Gender)	Median duration of disease in months (IQR unless specified)	Duration of follow-up (P)/JAKi use (R)	Concomitant medications (apart from steroids)	TN:TR	Previous therapies	Outcomes
Eriksson et al. [29]	Retrospective	TOF (1 pt), BAR (14 pt)	70.1 mean (range: 61−80)	15 (9F, 6M)	26.9 (mean) (range: 0−80)	Mean: 19 (range: 6−38)	No	All TR	GC+MTX 2 GC+TCZ 2 GC+TCZ, MTX, IFX 1	Clinical, ESR, CRP, GC dose
Loricera et al. [30]	Retrospective	BAR (15 pt), TOF (10 pt), UPA (10 pt)	72.3±8	35 (30, 5M)	30 (12, 48)	11 months (6-15.5), median (IQR)	One with MTX + steroids, 31 with steroids, 3 only JAKi	All TR	MTX 22 HCQ 3, LEF 1, TCZ 26* sarilumab 3^†^abatacept 8 adalimumab 2 ustekinumab 2	Clinical, ESR, CRP, GC dose
Koster et al. [11]	Prospective	BAR	72.4±7.2	15 (11F, 4M)	9 (7, 21)	52 weeks	No	All TR	GC alone 12 GC + MTX 1 GC + MTX, CYP 1 GC + sirukumab 1	Clinical/BVAS, PGA, ESR, CRP, GC dose
Camellino et al. [37]	Case report	BAR	Median: 69.5 (range: 60-83)	6 (5F, 1M)	33.1 (20.4, 80.8)	106.25 months (mean) (range: 88-126 months)	NS in 1 patient, none in 5 patients	NA	Pt 1: MTX, HCQ, SFZ; Pt 2: TCZ, MTX Pt 3: MTX, CYP, MMF, TCZ Pt 4: TCZ Pt 5: MTX, TCZ ^‡ ^Pt 6: MTX	Clinical, ESR, CRP, GC dose
Prigent et al. [38]	Case report	BAR	76	1F	2 years	12 months	NS	NA	MTX, TCZ	clinical, CRP, PET-CT
Blockmans et al. [39]	RCT	UPA	Placebo: 71.6 (7.3), UPA: 7.5 mg, 71.1 (7.5), UPA: 15 mg, 70.8 (7.3)	Placebo: 112 (77F, 35M), UPA; 7.5 mg, 107 (80F, 27M), UPA: 15 mg, 209 (156F, 53M)	(in days) Placebo TN: 37, TR: 277, UPA: 7.5 mg, TN: 3, TR: 539.5, UPA: 15 mg, TN: 36, TR: 343	52 weeks	No	Placebo: 67.9%:32.1%, UPA: 7.5: 70.1%:29.9%, UPA: 15: 70.8%:29.2%	NS	Clinical, ESR, CRP, GC dose

Outcomes of Takayasu Arteritis

The outcomes are summarized in Table 7.

**Table 7:** Takayasu arteritis study outcomes *Only statistically significant in the TN group; †Statistically significant difference; ‡No statistically significant difference TOF: tofacitinib; MTX: methotrexate; LEF: leflunomide; BAR: baricitinib; RUX: ruxolitinib; UPA: upadacitinib; INF: infliximab; CR: complete remission; LDSR: low-dose steroid remission; NS: not specified; SSD: statistically significant difference; HZ: herpes zoster; TN: treatment-naive; TR: treatment refractory: P10: prednisolone 10 mg daily; P15: prednisolone 15 mg daily; LCCA: left common carotid artery; RCCA: right common carotid artery; LSCA: left subclavian artery; NIH: National Institutes of Health

Study	Type	JAKi (dose)	Clinical	ESR CRP	Imaging	Relapse	GC dose	Adverse effect
Dai et al. [23]	Prospective	TOF: 5 mg BD	Improvement: 100%, CR: 94.1%	Reduced 100%	Stable: 82.3%, improvement: 17.6%	Nil	NS	NS
Kong et al. [24]	Prospective	TOF: 5 mg BD vs MTX (10-15 mg/wk)	TOF>MTX in CR and disease activity*, Use of TOF associated with CR at the 12th month*	ESR and CRP reduced with TOF not MTX*, ESR and CRP in TOF < MTX*	TN group: Improvement: TOF > MTX*, Overall and TR group: no SDD	TOF < MTX *, Relapse-free duration: TOF > MTX * all relapses in the MTX group are TN, all relapses in the TOF group are TR	TOF< MTX*	TOF: shingles 1 patient, MTX: liver derangement 3 patients (11.5%), loss of appetite 1 patient
Mv et al. [25]	Prospective	TOF: 5mg BD	Reduced clinical activity (SSD)	Reduced	NA	No	80% reduction to 5 mg/day	No
Wang et al. [26]	Prospective	TOF: 5 mg BD	Remission: LEF = TOF, LDSR: TOF > LEF† TN vs TR: ‡	CRP reduced in TOF† between TOF vs LEF: ‡ TR vs TN: ‡	Improvement TOF > LEF† TN vs TR: ‡	TOF = LEF, LEF grp: longer relapse-free duration†TOF pt who relapsed all TR	Both reduced vs baseline†LEF vs TOF: ‡, TOF > LEF achieve GC <7.5 mg/day†, TN grp: TOF < LEF TR grp: ‡	Higher in LEF, TOF: 2 cases of HZ, one increase in LDL
Zhou et al. [27]	Prospective	BAR: 4 mg daily	40% (4/10) maintained overall treatment response, 30% (3/10), no response	Reduced	80% (8/10) improvement/stabilization	1 patient at 12 months	80% (8/10) taper/maintain same dose, 2 stopped GC	Liver derangement: 1 patient
Li et al. [28]	Prospective	TOF 5 mg BD	80% (4/5) reduce symptoms and signs	80% (4/5) ESR CRP reduced	60% (3/5) reduced, 40% (2/5) same	NS	60% (3/5) same (P10 or P15), 40% (2/5) reduced	Nil
Yang et al. [31]	Case report	TOF: 10 mg/day	Remission NIH: 3-0	ESR CRP reduced	LCCA RCCA LSCA thickness reduced, aneurysm disappeared	Nil	Reduced	Infection
Palermo et al. [32]	Case report	TOF: 5 mg BD	Pulseless radial art pt 1, persistence of symptoms in pt 2	Increased	Pt 1: PET-CT reduced uptake, Pt 2: uptake increased	NS	NS	NS
Yamamura et al. [33]	Case report	TOF: 10 mg/day	Improved	Reduced	Improved	Nil	Reduced	Bacteremia of unknown origin, atheroma infection
Wang et al. [34]	Case report	Extended release TOF: 11 mg/day	Improved	Reduced	Improved	Nil	Reduced	Nil
Régnier et al. [16]	Case report	BAR: 4 mg/day x2 RUX: 10 mg/day x1 (also has primary polycythemia)	Remission NIH score: 3 to 0 x2, 1 to 0 x1	Reduced	NS	NS	Reduced x2 same x1	NS
Régent et al. [35]	Case report	BAR: 4 mg/day	Remission	Normalized	Improved	Nil	Tapered	Nil
Belfeki et al. [36]	Case report	UPA: 15 mg/day	Non-responsive until added INF	Normalized only after adding INF	Improved only after adding INF	Nil	Tapered	Two infections

Tofacitinib: In the prospective study by Dai et al. [23], levels of cytokines, chemokines, growth factors, and inflammatory markers were measured in 17 patients with TAK, which were then compared to 12 healthy controls in order to find out which signaling factors are associated with disease activity in TAK. Patients were treated with tofacitinib 5 mg BD for 12 months. The relevant factors, inflammatory markers, clinical activity, and MRA changes were re-evaluated. Tofacitinib use resulted in improvement in all patients, with 16 patients (94%) achieving CR and one patient partial remission (PR) at both six and 12 months. Fall in ESR and CRP levels was statistically significant at six months, but not at 12 months. MRA lesions stabilized in 14 patients (82%) at 12 months; improvement was seen in one patient at 12 months. Adverse effects during treatment were not mentioned.

Mv et al. [25] reported CR in eight out of 10 (80%) of refractory patients after tofacitinib. Disease activity was measured with the Indian Takayasu Arteritis Score 2010 (ITAS 2010) [46]. ESR and CRP both reduced at six months. Before tofacitinib, patients were on 10-20 mg of prednisolone or equivalent per day, with failure to taper steroids. After treatment, patients with CR were on prednisolone 5 mg/day. No adverse effects were found.

There are two studies in which tofacitinib was compared to other agents, namely, methotrexate and leflunomide. In the study by Kong et al. [24], in which tofacitinib was compared with methotrexate, 27 patients were allocated to the tofacitinib group, and 26 patients were in the methotrexate group. Baseline characteristics were comparable, except that the proportion of treatment-refractory patients is significantly higher in the tofacitinib group than in the methotrexate group. The CR rate was higher in the tofacitinib group at 12 months. A logistic regression analysis of baseline ESR levels, treatment group, systemic symptoms presence, and treatment history was done to see which factors were associated with CR at 12 months. It showed that lower baseline ESR levels, use of tofacitinib, and the presence of systemic symptoms were associated with CR at 12 months. However, other differences between the two groups were not analyzed. Reduction of ESR and CRP was only statistically significant in the tofacitinib group, with ESR and CRP levels being significantly lower in the tofacitinib group. The median glucocorticoid dose in the tofacitinib group was also significantly lower than that in the methotrexate group from the third month onwards, and more patients in the tofacitinib group were on prednisolone <7.5 mg/day. Relapse-free duration was longer in the tofacitinib group. Further subgroup analysis between treatment naive and treatment refractory patients revealed, however, that findings were statistically significant only in the treatment naive group. All eighty cases of relapse in the methotrexate group occurred in treatment-naive patients, while all three relapses in the tofacitinib group happened in refractory patients. For adverse effects, one patient taking tofacitinib had herpes zoster, which resolved after temporarily withholding tofacitinib for two weeks and acyclovir treatment. For the methotrexate group, three patients had raised liver enzyme levels higher than two-thirds of the upper limit of normal. All resolved with glutathione and without stopping methotrexate. One patient had loss of appetite at initiation of treatment, which later then resolved spontaneously.

Another prospective study by Wang et al. [26] compared tofacitinib with leflunomide. The tofacitinib group (consisting of 32 patients) had a statistically higher proportion of treatment-refractory patients and patients with longer duration of disease, while baseline glucocorticoid dosage was higher in the leflunomide group (35 patients). There was no significant difference between the two groups in terms of effectiveness rate (clinical remission rate plus PR rate, but the low-dose glucocorticoid remission (LDGR, defined as persistent remission from six months to 12 months and achievement of GCs ≤7.5 mg/day at 12 months) was higher in the tofacitinib group. Among factors such as treatment group, sex, age, disease duration, GCs dose, ESR, CRP levels, TN cases, and TR cases, a binary regression analysis showed that TOF treatment, chest pain/distress, and the GCs dose at baseline were associated with LDGR, but not CR, at 12 months. Relapse prevalence was comparable between the two groups, but patients who took leflunomide had a longer relapse-free duration. As for ESR and CRP levels, CRP levels reduced throughout the 12 months compared to baseline in the tofacitinib group, but not in the leflunomide group. There was no significant change in ESR levels in the tofacitinib group at 12 months. When the two treatment groups were compared, there was no statistically significant difference in the levels of ESR and CRP. The proportion of patients with a daily glucocorticoid dose ≤7.5 mg or 5 mg was significantly higher in the tofacitinib group, but the leflunomide group had a higher baseline dosage of glucocorticoids. A higher proportion of patients in the tofacitinib group had improvement on imaging studies at 12 months. When treatment-naive and treatment-refractory patients were analyzed separately, there was no significant difference between the two treatment groups in terms of effectiveness rate. Among treatment-naive patients, the tofacitinib group had significantly lower glucocorticoid dosage compared to the leflunomide group at 12 months. Among treatment-refractory patients, absolute glucocorticoid dosages were lower in the tofacitinib group at baseline and throughout the study till 12 months, but further analysis did not reveal any significant difference in the percentage reduction of glucocorticoid use between the tofacitinib and leflunomide groups. There were also no significant differences observed in changes in ESR, CRP, and imaging between the two treatment groups when treatment-naive and treatment-refractory patients were analyzed separately. The prevalence of side effects in the leflunomide group was found to be higher than that in the tofacitinib group, but the infection rates were comparable.

Li et al. [28] followed five patients refractory to disease-modifying antirheumatic drugs (DMARDs) and biologics for at least six months after being started on tofacitinib 5 mg BD. At six months, four out of five patients (80%) of patients had improvement in clinical symptoms and inflammatory markers. Three out of five patients (60%) had improvement in imaging findings, while the other two had more or less static lesions. Two patients (40%) reduced corticosteroid dosage: one from prednisolone 15 mg daily to 10 mg daily, and the other from 27.5 mg daily to 10 mg daily. The other three patients were kept on the same dose of prednisolone (15 mg daily or 10 mg daily). No adverse effects were experienced.

For case reports, a total of four patients were reported. All are refractory to at least two immunosuppressants, and eight patients had received tocilizumab. Follow-up time ranged from two months to 33 months. Only one [34] reported the use of extended-release tofacitinib. One patient temporarily had mycophenolate, tocilizumab, and leflunomide with tofacitinib [31], and another patient [33] had combination therapy with methotrexate. Three patients [31,33,34] had improvement in clinical activity, inflammatory markers, steroid requirement, and/or imaging findings. Two patients remained refractory to tofacitinib [32]. Adverse effects were not mentioned in one report [32]. Two patients [31,33] developed infections while taking tofacitinib, and the other patients didn’t suffer from any side effects.

Baricitinib: The prospective study by Zhou et al. [27], 10 patients with TAK refractory to steroids, and at least two traditional immunosuppressants or biologics were started on baricitinib 4 mg/day. Eight patients received one other concurrent traditional immunosuppressant (methotrexate or leflunomide or hydroxychloroquine), while two had it together with methotrexate and hydroxychloroquine, with no dose adjustments. At the end of the study, three out of 10 patients (30%) were complete responders, one (10%) a partial responder, and the remaining six (60%) were non-responders. Apart from two patients who stopped baricitinib at four months due to deterioration in vascular lesions, all other patients (eight out of 10 (80%)) had improvement or stabilization of vascular lesions on CTA and Doppler ultrasound. Eight patients reduced or maintained steroid dosage, two of whom were able to stop steroids at 18 and 24 months, respectively, and stayed in remission till the end of the study. Some of the non-responders might have had more difficult-to-control disease at baseline. Two of them required methylprednisolone at baseline, while one failed eight different immunosuppressants, of which three were biologics of different classes. One patient with CR had elevated liver enzymes 18 months after starting baricitinib. Liver function returned to normal after baricitinib was stopped. There were no other adverse effects experienced.

The two case reports on baricitinib involved patients whose disease was refractory to at least three immunosuppressants, including biological agents, such as tocilizumab and TNF inhibitors. Follow-up time was six months. All of the patients achieved good disease control with baricitinib, with normalization of inflammatory markers, and maintenance or reduction of steroid doses. There is no information regarding imaging findings or side effects in the report by Régnier et al. [16], while the patient reported by Régent et al. [35] had resolution of imaging abnormalities and did not experience any side effects.

Ruxolinitib: The only report of ruxolitinib use in TAK was by Régnier et al. [16], in which a male patient who had concomitant primary polycythemia was treated with ruxolitinib 10 mg/day. He was treatment-naive. After ruxolitinib, the NIH score reduced from three to zero, and the steroid dose and CRP level were reduced.

Upadacitinib: Belfeki et al. [36] reported a case of a 33-year-old female who failed steroid and tocilizumab therapy. Her disease was still not well controlled after switching from tocilizumab to infliximab and methotrexate. Infliximab was then changed to upadacitinib, but the patient did not respond after six months. Her disease could only be controlled after the addition of infliximab to methotrexate and upadacitinib. She had two episodes of infection treated with antibiotics.

Outcomes of Giant Cell Arteritis

The results for GCA are summarized in Table 8.

**Table 8:** GCA study outcomes * clinical improvement (baricitinib (n=7; 44%), tofacitinib (n=5; 31%), upadacitinib (n=4; 25%)); complete remission (baricitinib (n=6; 46%), tofacitinib (n=3; 23%), upadacitinib (n=4; 31%)); † five were on tofacitinib, four on baricitinib, and two on upadacitinib; ‡ five patients were receiving tofacitinib, two baricitinib, and two upadacitinib; §PMR (2pt): remission x1 improvement x1, PMR+GCA (2pt): remission x1, improvement x1, GCA (2pt): remission x1, relapse (remitted with TCZ); ¶ Sustained remission rate: absence of signs or symptoms of giant-cell arteritis from week 12 through week 52 and adherence to the protocol-specified glucocorticoid taper; # Sustained complete remission: sustained remission with normalization of the ESR and the CRP level from week 12 through week 52 PMR: polymyalgia rheumatica; GCA: giant cell arteritis; JAKi: JAK inhibitors; TOF: tofacitinib; UPA: upadacitinib; BAR: baricitinib; NS: not specified; NA: not applicable; Pt: patient; UTI: urinary tract infection; dLFT: deranged liver function tests; SOB: shortness of breath; HZ: herpes zoster; LDL: low-density lipoprotein; HDL: high-density lipoprotein; MACE: major adverse cardiac events; UPA7.5: upadacitinib 7.5 mg daily; UPA15: upadacitinib 15 mg daily; VTE: venous thromboembolism; CK: creatine kinase; RCT: randomized controlled trial; HTN: hypertension; NSC: nonmelanoma skin cancer

Study	Type	JAKi (dose)	Clinical	ESR CRP	Imaging	Relapse	GC dose	Adverse effect
Eriksson et al. [29]	Retrospective	BAR 2−4 mg, TOF 10mg/d	73% remission at 6 months	Reduced	NS	Nil	Reduced	Enterococcus bacteremia (tofacitinib), aspergillus infection (baricitinib)
Loricera et al. [30]	Retrospective	15 (43%) BAR (2–4 mg daily) 10 (29%) TOF (5 mg BD 10 (29%) UPA (15 mg daily)	Improvement 70% at 12 months. Remission 65% at 12 months, similar across all JAKi. At 18m CR: TOF 80%, UPA 50%. At 24m CR, TOF 50%. Anti-IL6 before (n=28) 57% improvement. 46% complete remission*	Reduced Pt with IL6 before: insignificant difference	NS	31% relapse/ persistence of active disease^.†^ Anti-IL6 before: Relapse in 32%^ ‡^	Reduced 20% stop steroids. With IL6 before: reduced, 24% stopped	BAR: UTI-1 dLFT-1. TOF: Palpitations/SOB-1. UPA: Disseminated HZ-1, glioblastoma multiforme-1
Koster et al. [11]	Prospective	BAR: 4 mg/day	Remission: 93%	Reduced	NA	1 of 14 (7%)	Reduced: 93% stopped steroids	Infection not requiring antibiotics (n=8). Infection requiring antibiotics (n=5). Nausea (n=6). Leg swelling (n=2). Fatigue (n=2). Diarrhea (n=1). Abdominal pain (n=1), HZ (n=1), transient thrombocytopenia (n=1), LDL increased, HDL decreased, no MACE
Camellino et al. [37]	Case report	BAR: 4 mg/day	Remission: 50%. Improvement: 33%. Relapse: 17% ^§ ^	NS	GCA Pt who relapsed: no improvement. Improvement in pts with GCA+PMR. Others: NS	1 (pt with GCA)	All reduced: 33% patients stopped GC	Pneumonia (1 pt)
Prigent et al. [38]	Case report	BAR: 4 mg/day	Remission	CRP reduced	Resolved	Nil	Stopped	NS
Blockmans et al. [39]	RCT	Placebo vs UPA 7.5 mg/d vs 15 mg/day	Sustained remission rate¶ (%): Placebo: 29.0. UPA: 7.5, 41.1. UPA: 15, 46.4	Sustained complete remission rate# (%): Placebo: 16.1. UPA: 7.5, 26.2. UPA: 15, 37.1	NA	Till week 52 (%): Placebo: 55.6. UPA: 7.5, 41.3. UPA: 1534.3	Median cumulative dose till week 52 (mg): Placebo: 2882. UPA: 7.5, 1905. UPA: 15, 1615	UPA15: headache 16.3%, arthralgia 13.9%, HTN 13.4%, Covid-19 13.4%. UPA15 vs placebo: (%). Serious infection (5.7 vs 10.7), HZ (5.3 vs 2.7). Cancer except NSC (1.9 vs 1.8), NSC (2.4 vs 1.8), MACE (0 vs 1.6), VTE (3.3 vs 3.6), dLFT (5.3 vs 4.5), CK elevated (2.9 vs 0), Anemia (6.7 vs 0), lymphopenia (1.4 vs 0)

In the retrospective study by Eriksson et al. [29], 14 patients who failed corticosteroid therapy and/or tocilizumab, or whom tocilizumab was considered inappropriate, were started on baricitinib 2-4 mg/day, and one patient was started on tofacitinib 10 mg/day. They were on JAKi for at least six months. Data from the latest follow-up, six months after, three months after, and at initiation of treatment were analyzed. Five patients had immunosuppressants, such as methotrexate, tocilizumab, or infliximab before, and none had concomitant therapies other than steroids with JAKi. Overall, 73% of patients achieved the primary outcome of “therapeutic benefit” at six months. There was a significant reduction of CRP levels at three months, six months, and at last follow-up, while ESR reduced significantly at six months and at last follow-up. Statistically significant decrease in prednisolone dose was also seen at three months, six months, and at the last follow-up. There was no relapse while patients were on JAK inhibitors. The patient on tofacitinib had similar ESR levels at baseline and last follow-up, but clinical activity, CRP, and steroid dose were all reduced. Three patients stopped baricitinib due to sustained remission after two years. However, one of them had a relapse, which was then controlled when baricitinib was reintroduced. Two patients suffered from severe infections, one of which was an *Aspergillus fumigatus* infection and the other was *Enterococcus faecalis *bacteremia. The patient who had an *Aspergillus *infection already had suspicious lung nodules before starting baricitinib. One of the nodules started to cavitate after baricitinib was started, and investigations revealed growth of *A. fumigatus*. He was treated with antifungal without cessation of baricitinib. The patient who was started on tofacitinib developed *E. faecalis *bacteremia from biliary infection, which was treated with antibiotics and temporary cessation of tofacitinib.

In another study by Loricera et al. [30], 35 patients were treated with JAKi: 15 with baricitinib, 10 with tofacitinib, and 10 with upadacitinib. Patients were followed up for a median of 11 months, with all patients followed up for at least one month, 33 patients for at least three months, 28 patients followed up for at least six months, and 20 patients followed up for at least 12 months. No patients were on baricitinib for at least 18 months, while five were on tofacitinib and two on upadacitinib for at least 18 months. Two patients were on tofacitinib and one on upadacitinib for at least 24 months. Overall, clinical remission was seen in 70% of all patients, and CR was seen in 65% at 12 months, with results being comparable among the three agents. Overall, median ESR declined as compared to baseline at first, sixth, 12th, 18th month, and last follow-up. Analyzed separately, the decline was significant for baricitinib through the 3rd-12th month. Overall, there was no significant fall in CRP from baseline to last follow-up, but CRP levels fell significantly from one month to 12 months for baricitinib. The median daily dose of prednisone decreased from 16.2 mg to 5 mg at the last follow-up, with 20% of patients stopping glucocorticoids. Prednisolone daily dose all reduced significantly at months one, six, and 12 for each treatment subgroup. The reduction in steroids remained significant at 18 months for tofacitinib and upadacitinib and at 24 months for tofacitinib. Twenty-eight (80%) patients had previously received anti-IL6 agents, 25 had tocilizumab alone, two had sarilumab alone, and one had both. It was stopped due to inefficiency in 20 of them. Of the 28 patients, 10 received baricitinib, nine tofacitinib, and nine upadacitinib. Additionally, 60% of the patients on baricitinib, 33% on tofacitinib, and 44% on upadacitinib achieved CR. Among patients who used anti-IL6 agents before, there was no significant reduction in ESR and CRP, but values were already normal at initiation. Overall, the prednisone dose decreased from a median of 15.6 mg/day to 5 mg/day at the last follow-up, and 21% of these patients managed to discontinue steroids. Fifty percent of patients on tofacitinib and 20% of patients on upadacitinib stopped JAKi due to relapse or refractory disease. All of them had anti-IL6 therapy before. Twenty-seven percent of patients on baricitinib stopped JAKi, and half of them had anti-IL6 before. Five patients developed adverse events. A patient had a urinary tract infection, but could continue baricitinib. The other four patients had to stop JAKi permanently. A patient on baricitinib developed a significant elevation of liver enzymes, and another on tofacitinib developed palpitations and dyspnea. One patient on upadacitinib had disseminated herpes zoster, and one was diagnosed with glioblastoma multiforme six months after starting upadacitinib.

In the prospective study by Koster et al. [11], 15 patients with relapsing GCA were started on baricitinib 4 mg/day. Patients were on different doses of steroids at the start of baricitinib, ranging from 10 mg/day to 30 mg/day. One patient had to stop baricitinib at week eight due to renal impairment. Apart from one patient who relapsed when steroids were tapered, all the other patients tapered off steroids and successfully remained in remission till the end of the study period. Mean levels of ESR, CRP, patient global assessment, and BVAS [47] all significantly reduced at week 24 and week 52 compared to baseline. Four patients flared in the 12-week follow-up period after baricitinib discontinuation. Although there were no major adverse cardiac events, it was noted that mean LDL levels increased and HDL levels decreased at week 16 compared to baseline, but no patients needed to start lipid-lowering therapy. One patient developed herpes zoster; thus, baricitinib was withheld for two weeks, and the patient was treated with acyclovir. The patient subsequently continued baricitinib for 32 more weeks. Fourteen out of 15 patients had at least one minor adverse effect, including infection not requiring antibiotics, infection requiring antibiotics, nausea, leg swelling (without venous thromboembolism), fatigue, diarrhea, and abdominal pain.

Camellino et al. [37] reported six patients with refractory or relapsing PMR and/or GCA who took baricitinib; two patients had PMR without vasculitis, two presented with PMR and were found to have large vessel vasculitis, and two had GCA without many PMR symptoms. Three out of six patients (one with PMR, one with PMR/GCA, and one with GCA) were started on baricitinib because of the shortage of tocilizumab during the COVID pandemic. Two of them (one with GCA, one with PMR/GCA) were previously on tocilizumab with good disease control. These two patients were later switched back to tocilizumab, as baricitinib was not effective in the patient with GCA, and the other patient with PMR/GCA, although responsive to baricitinib, had a lung infection. The other four patients had a good clinical response with baricitinib and could taper down steroids. One patient could stop steroids without relapse.

Prigent et al. [38] reported a case of a 76-year-old woman whose GCA was refractory to methotrexate and tocilizumab. She was started on baricitinib, and after six months, her clinical symptoms and CRP levels improved, and PET-CT showed resolved vasculitic changes. Twelve months after starting baricitinib, she could stop steroids.

Blockmans et al.'s [39] study is so far the only RCT on JAKi in large vessel vasculitis. Patients were randomized into receiving upadacitinib 15 mg daily, upadacitinib 7.5 mg daily, and placebo on a 2:1:1 ratio. Patients with upadacitinib were put on a prespecified 26-week glucocorticoid taper schedule, while those in the placebo group had a prespecified 52-week glucocorticoid taper. The study excluded patients who had JAKi before, or whose disease was not controlled with anti-IL6. Initially, 428 patients were recruited, but only 299 of them completed the regimen through 52 weeks. Baseline characteristics between the groups are roughly the same. In all three groups, around 70% of patients had new onset GCA, while 30% had relapsing disease. Sustained remission rate and sustained CR rate were significantly higher in the upadacitinib 15 mg/day group than the placebo group, while a significant difference was not observed in the upadacitinib 7.5 mg/day group. Superiority of upadacitinib 15 mg/day over placebo was observed in different subgroups, including age, sex, new-onset or relapsing GCA, and baseline glucocorticoid dose. Cumulative glucocorticoid dosage in the upadacitinib 15 mg/day group was lower than the placebo group, with more patients achieving remission without any steroids. The risk of flare in patients on upadacitinib 15 mg/day is also lower. There was also a significant reduction in patient-reported fatigue and quality of life in the upadacitinib 15 mg/day group than the placebo group. For side effects, the most common ones in patients taking upadacitinib 15 mg/day were headache, arthralgia, hypertension, and COVID-19. Adverse events leading to discontinuation of upadacitinib or placebo were higher in the placebo group (19.6%) than in the upadacitinib 15 mg/day group (14.8%). Compared with placebo, there was no increased risk of major adverse cardiovascular events, thromboembolism, or serious infection. However, the risk of opportunistic infections, herpes zoster, non-melanoma skin cancer, hepatic disorder, anemia, lymphopenia, elevation of creatine kinase, and bone fracture was higher in the upadacitinib 15 mg/day group.

Phase two of the study will further investigate the safety and efficacy of continuing upadacitinib in patients who achieved remission in phase one.

CR was defined to satisfy four criteria: (1) no new/worsened systemic symptoms; (2) no new/worsened vascular symptoms or signs; (3) erythrocyte sedimentation rate (ESR) was normal (≤40 mm/hour); and (4) GC dose ≤15 mg/day.

PR was defined as two combined with any two of the criterion above: 1, 3, or 4. Relapse was denoted as reactivation of disease activity (NIH criteria ≥2 points) for patients who had achieved CR or PR.

CR was defined as the absence of disease activity (ITAS 2010 = 0); PR was defined as ITAS 2010 = 1; and (3) persistently active disease was defined as inability to attain ITAS 2020 = 1 or persistently raised CRP after four to six weeks of treatment. We defined relapse as any new clinical manifestations or increase in ITAS after achieving CR or PR during the study.

CR was defined as (i) no new symptoms or worsening of systemic symptoms; (ii) no new symptoms or worsening of vascular symptoms or signs; (iii) normal ESR (≤40 mm/hour); and (iv) GC dose ≤15 mg/day (six months) or ≤10 mg/day (12 months). PR was defined as meeting criterion (ii) combined with any two of (i), (iii), and (iv). ER was considered if patients achieved CR or PR. “Relapse” indicated reactivation of disease (NIH criteria ≥2 points) after patients had achieved CR or PR.

Complete response was defined as no evidence of active disease: (1) ESR <20 mm/hour and CRP <10 mg/L, (2) no progression of vessel damage, and (3) the dose of GC <15 mg/day prednisone (or equivalent). A PR was defined as (1) ESR <40 mm/hour or decrease over 50% compared with baseline, (2) CRP <20 mg/L or decrease over 50% compared with baseline, and (3) not fulfilling the other two criteria of CR.

Relapses were defined according to 2018 European League Against Rheumatism (EULAR) recommendations: the presence of typical signs or symptoms of TAK with at least one of the following: (1) current activity on imaging or biopsy; (2) ischaemic complications attributed to TAK; or (3) persistently elevated inflammatory markers, excluding other causes

Therapeutic benefit was defined as without suspicion of clinical activity and with the absence of new GCA symptoms (suspected to be related to GCA) and decreasing or stable CRP combined with decreasing daily prednisolone dose at three and six months post-initiation of JAKi.

Relapse of GCA was defined as suspicion of clinical activity based on relevant symptoms and increasing CRP (in the absence of other concomitant diseases), leading to an increase in the daily prednisolone dose.

Clinical remission was defined as the absence of signs and symptoms attributable to GCA (e.g., headaches, jaw claudication, polymyalgia rheumatica symptoms (PMR), etc.) regardless of the value of the ESR and CRP.

CR was defined, as per the EULAR criteria, as the absence of signs and symptoms attributable to GCA and the normalization of the ESR and CRP values.

Relapse was defined as the reappearance of clinical manifestations of GCA that required treatment intensification.

Discussion

This study corroborates the findings of a similar systematic review by Rathore et al. [22], which also concludes that JAKi are promising agents in the treatment of large vessel vasculitis. More studies have since then been carried out and are included here. This review excludes reports in which patients have concomitant autoimmune diseases, since this may be a significant factor affecting response to JAKi and the choice of JAKi used. Most studies and reports show favorable results for JAKi. Most of the evidence for JAKi in large vessel vasculitis is for TAK, with the majority of them being case reports. Tofacitinib is by far the most studied, followed by baricitinib. All patients described in case reports are refractory to at least two immunosuppressants other than steroids. Apart from the two patients reported by Palermo et al. [32] and one patient described by Li et al. [28], other patients in case reports had favorable clinical responses to tofacitinib. There are so far three cohort studies with tofacitinib, with two that compare tofacitinib with traditional immunosuppressants, namely, methotrexate and leflunomide. Tofacitinib seems to be effective in reducing clinical activity, achieving clinical remission, and preventing relapse, but it may not be superior to leflunomide with regard to these outcomes. Tofacitinib is superior to methotrexate and leflunomide in improving imaging abnormalities, reducing daily steroid dose, and maintenance of remission at low steroid dose. Although the case reports, being all about treatment-refractory cases, may suggest that tofacitinib is effective in treatment-refractory patients, the two comparative studies reveal that it may not be particularly more effective than methotrexate or leflunomide in these patients.

For baricitinib, the two case reports and the cohort study suggest that it is useful in improving clinical outcomes in refractory TAK, including reducing clinical activity, inflammatory marker levels, imaging findings, and reducing steroid requirements. The only reported use of ruxolitinib was in a patient with primary polycythemia, which may be the reason why ruxolitinib was the chosen agent, as it is an approved agent in the treatment of primary polycythemia. Although the patient responded well, the results are to be interpreted carefully since his concomitant condition can be a significant confounding factor.

As for GCA, there are three cohort studies on the use of baricitinib, upadacitinib, and tofacitinib, two case reports on baricitinib, and one double blind RCT on upadacitinib. JAKi appears to be effective in achieving improvement in terms of clinical activity, inflammatory marker levels, steroid requirement, and relapse. In the retrospective study, the efficacy of the three different agents is similar at 12 months, but the follow-up time and duration of use of JAKi vary quite a lot among the patients, from as short as one month with baricitinib to 24 months with tofacitinib and upadacitinib. It is therefore not known what the clinical outcome may be if patients with shorter follow-up/JAKi therapy durations are followed up longer. The RCT provides stronger evidence of the usefulness of upadacitinib 15 mg/day, but not upadacitinib 7.5 mg/day, over placebo in the treatment of GCA. However, the study does not mention whether patients with refractory disease were put on disease-modifying agents before. Patients who failed JAKi or anti-IL6 before were also excluded. Therefore, the effectiveness of upadacitinib in patients who have difficult-to-control GCA and who fail other steroid-sparing therapies or biologics remains to be further elucidated.

With regard to side effects, JAKi are, in general, well-tolerated. In the two comparative studies, JAKi are associated with fewer adverse effects than methotrexate and leflunomide. The most common side effect is infection, most of which can be settled by appropriate antimicrobials and temporary cessation of JAKi. The risk of herpes zoster is indeed observed to be higher. As for gastrointestinal perforation, major adverse cardiovascular events, and venous thromboembolism, no such events have been identified in the included studies. The RCT showed a slightly higher risk of cancer, anemia, and lymphopenia among users of upadacitinib, but only one case of cancer (glioblastoma multiforme) and no cases of blood dyscrasias have been reported in the observational studies and case reports.

Although the studies are of reasonable quality, most of them are case reports, and most of the cohort studies lack a control group. Sample sizes are also quite small. The two studies that compare tofacitinib with methotrexate and leflunomide are limited by baseline differences, such as the proportion of treatment-refractory to treatment-naive patients (with tofacitinib having more treatment-refractory patients), disease duration, etc. The RCT excluded patients whose disease was more severe and failed previous biologics. Larger studies with comparable control groups are needed to further confirm the utility of JAKi in TAK and GCA, particularly in difficult-to-treat cases. The effectiveness of the different JAKi may also be further investigated and compared, as they have slightly different molecular targets. There are currently a number of future or ongoing clinical trials that compare JAKi to other therapies. The ongoing/future trials are summarized in Table 9.

**Table 9:** Ongoing/future trials DB: double blinded; RCT: randomized control trials; TAK: Takayasu arteritis; GCA: giant cell arteritis

Study name	Drug (control)	Condition	Type	Recruitment	Current Status
SELECT-TAK	Upadacitinib (Placebo)	TAK	DB, RCT	56 (Estimated)	Active, not recruiting
ADATOFTAK	Upadacitinib (Adalimumab)	GCA	RCT	100 (Estimated)	Recruiting
Comparison of Tofacitinib and Methotrexate in Takayasu's Arteritis	Tofacitinib (Methotrexate)	TAK	RCT	76 (Estimated)	Recruiting
TOFGCTAK	Tofacitinib (Prednisolone)	TAK	DB, RCT	50 (Estimated)	Recruiting

## Conclusions

This review shows that JAKi are potentially effective therapeutic agents in TAK and GCA. Their side effects are also, in general, well-tolerated. Case reports demonstrate that JAKi may be efficacious in treatment-refractory patients, but comparative studies suggest that they may not be superior to traditional agents.

This study has several limitations. Firstly, studies included are mostly case reports, and most observational studies do not have a control group. Secondly, the definitions of clinical remission, refractoriness, or relapse vary across different studies, which may affect the fairness of comparison. There is therefore a need for more robust, universal definitions for disease remission, relapse, and refractory diseases, which should be adapted across different studies. There is so far only one double-blinded RCT comparing upadacitinib with placebo. More RCTs with adequate sample size are needed to better delineate the therapeutic potential of JAKi in large vessel vasculitis, particularly in treatment-refractory patients. The effectiveness of the different JAKi, and their effectiveness as compared to currently used disease-modifying agents and biologics, can be further investigated.

## Appendices

**Table 10:** Search strategy

Database	Keywords	Search strategy	Results before filter	Filters	After filter
PubMed	Takayasu arteritis, giant cell arteritis, JAK inhibitors, tofacitinib, upadacitinib, baricitinib, ruxolitinib, effectiveness, evidence, efficacy, safety, use	(( "Giant Cell Arteritis/classification"[Mesh] OR "Giant Cell Arteritis/diagnosis"[Mesh] OR "Giant Cell Arteritis/diagnostic imaging"[Mesh] OR "Giant Cell Arteritis/immunology"[Mesh] OR "Giant Cell Arteritis/therapy"[Mesh] )) AND ( "Janus Kinase Inhibitors/adverse effects"[Mesh] OR "Janus Kinase Inhibitors/classification"[Mesh] OR "Janus Kinase Inhibitors/immunology"[Mesh] OR "Janus Kinase Inhibitors/therapeutic use"[Mesh] ) AND (effectiveness OR safety OR evidence OR efficacy OR use)	6	within 5 years Case Reports Clinical Study Clinical Trial Clinical Trial Protocol Clinical Trial, Phase I Clinical Trial, Phase II Clinical Trial, Phase III Clinical Trial, Phase IV Controlled Clinical Trial Meta-Analysis Multicenter Study Observational Study Randomized Controlled Trial Review Systematic Review	5
		(((giant cell arteritis) ) AND (JAK inhibitors)) AND (effectiveness OR safety OR evidence OR efficacy OR use)	26		14
		(((giant cell arteritis) ) AND (JAK inhibitors))	31		18
		(((giant cell arteritis) ) AND (baricitinib)) AND ((effectiveness OR safety OR evidence OR efficacy OR use)	9		6
		(((giant cell arteritis) ) AND (baricitinib))	10		6
		(((giant cell arteritis) ) AND (upadacitinib)) AND (effectiveness OR safety OR evidence OR efficacy OR use)	8		6
		(((giant cell arteritis) ) AND (upadacitinib))	9		6
		(((giant cell arteritis) ) AND (tofactinib)) AND (effectiveness OR safety OR evidence OR efficacy OR use)	11		7
		(((giant cell arteritis) ) AND (tofactinib))	14		8
		(((giant cell arteritis) ) AND (ruxolitinib)) AND (effectiveness OR safety OR evidence OR efficacy OR use)	2		1
		(((giant cell arteritis) ) AND (ruxolitinib))	2		1
		((giant cell arteritis) ) AND (jak)	37		23
		( "Takayasu Arteritis/diagnosis"[Mesh] OR "Takayasu Arteritis/diagnostic imaging"[Mesh] OR "Takayasu Arteritis/immunology"[Mesh] OR "Takayasu Arteritis/therapy"[Mesh] ) AND ( "Janus Kinase Inhibitors/adverse effects"[Mesh] OR "Janus Kinase Inhibitors/classification"[Mesh] OR "Janus Kinase Inhibitors/immunology"[Mesh] OR "Janus Kinase Inhibitors/therapeutic use"[Mesh] ) AND (effectiveness OR safety OR evidence OR efficacy OR use)	5		5
		((Takayasu Arteritis OR Takayasu’s Arteritis) ) AND (JAK inhibitors)) AND (effectiveness OR safety OR evidence OR efficacy OR use)	19		11
		((Takayasu Arteritis OR Takayasu’s Arteritis) ) AND (JAK inhibitors))	33		18
		((Takayasu Arteritis OR Takayasu’s Arteritis) ) AND (jak)	19		13
		((Takayasu Arteritis OR Takayasu’s Arteritis) ) AND (baricitinib)) AND ((effectiveness OR safety OR evidence OR efficacy OR use)	5		4
		((Takayasu Arteritis OR Takayasu’s Arteritis) ) AND (baricitinib))	5		4
		((Takayasu Arteritis OR Takayasu’s Arteritis) ) AND (upadacitinib)) AND ((effectiveness OR safety OR evidence OR efficacy OR use)	3		2
		((Takayasu Arteritis OR Takayasu’s Arteritis) ) AND (upadacitinib)	4		3
		((Takayasu Arteritis OR Takayasu’s Arteritis) ) AND (tofacitinib)) AND ((effectiveness OR safety OR evidence OR efficacy OR use)	12		6
		((Takayasu Arteritis OR Takayasu’s Arteritis) ) AND (tofacitinib))	26		13
		((Takayasu Arteritis OR Takayasu’s Arteritis) ) AND (ruxolitinib)) AND ((effectiveness OR safety OR evidence OR efficacy OR use)	2		1
		((Takayasu Arteritis OR Takayasu’s Arteritis) ) AND (ruxolitinib))	2		1
		"JAK inhibitors" OR baricitinib OR upadacitinib OR tofacitinib OR ruxolitinib) AND "large vessel vasculitis"	12		12
		"JAK inhibitors" OR baricitinib OR upadacitinib OR tofacitinib OR ruxolitinib) AND "large vessel vasculitis" AND ((effectiveness OR safety OR evidence OR efficacy OR use)	15		10
PMC	Takayasu arteritis, giant cell arteritis, JAK inhibitors, tofacitinib, upadacitinib, baricitinib, ruxolitinib, effectiveness, evidence, efficacy, safety, use	(( "Giant Cell Arteritis/classification"[Mesh] OR "Giant Cell Arteritis/diagnosis"[Mesh] OR "Giant Cell Arteritis/diagnostic imaging"[Mesh] OR "Giant Cell Arteritis/immunology"[Mesh] OR "Giant Cell Arteritis/therapy"[Mesh] )) AND ( "Janus Kinase Inhibitors/adverse effects"[Mesh] OR "Janus Kinase Inhibitors/classification"[Mesh] OR "Janus Kinase Inhibitors/immunology"[Mesh] OR "Janus Kinase Inhibitors/therapeutic use"[Mesh] ) AND (effectiveness OR safety OR evidence OR efficacy OR use)	5	2019-2024 Title	4
		(((giant cell arteritis) ) AND (JAK inhibitors)) AND (effectiveness OR safety OR evidence OR efficacy OR use)	711		0
		(((giant cell arteritis) ) AND (JAK inhibitors))	728		0
		(((giant cell arteritis) ) AND (baricitinib)) AND ((effectiveness OR safety OR evidence OR efficacy OR use)	328		0
		(((giant cell arteritis) ) AND (baricitinib))	357		1
		(((giant cell arteritis) ) AND (upadacitinib)) AND (effectiveness OR safety OR evidence OR efficacy OR use)	149		0
		(((giant cell arteritis) ) AND (upadacitinib))	143		0
		(((giant cell arteritis) ) AND (tofacitinib)) AND (effectiveness OR safety OR evidence OR efficacy OR use)	395		0
		(((giant cell arteritis) ) AND (tofacitinib))	368		0
		(((giant cell arteritis) ) AND (ruxolitinib)) AND (effectiveness OR safety OR evidence OR efficacy OR use)	194		0
		((giant cell arteritis) ) AND (ruxolitinib))	199		0
		( "Takayasu Arteritis/diagnosis"[Mesh] OR "Takayasu Arteritis/diagnostic imaging"[Mesh] OR "Takayasu Arteritis/immunology"[Mesh] OR "Takayasu Arteritis/therapy"[Mesh] ) AND ( "Janus Kinase Inhibitors/adverse effects"[Mesh] OR "Janus Kinase Inhibitors/classification"[Mesh] OR "Janus Kinase Inhibitors/immunology"[Mesh] OR "Janus Kinase Inhibitors/therapeutic use"[Mesh] ) AND (effectiveness OR safety OR evidence OR efficacy OR use)	2		2
		((Takayasu Arteritis OR Takayasu’s arteritis) ) AND (“JAK inhibitors” OR “januse kinase inhibitors”)) AND (effectiveness OR safety OR evidence OR efficacy OR use)	138		0
		((Takayasu Arteritis OR Takayasu’s arteritis) ) AND (“JAK inhibitors” OR “januse kinase inhibitors”))	140		0
		(Takayasu Arteritis OR Takayasu’s arteritis) ) AND (baricitinib)) AND ((effectiveness OR safety OR evidence OR efficacy OR use)	122		0
		((Takayasu Arteritis OR Takayasu’s arteritis) ) AND (baricitinib))	124		1
		((Takayasu Arteritis OR Takayasu’s arteritis) ) AND (upadacitinib)) AND ((effectiveness OR safety OR evidence OR efficacy OR use)	65		0
		((Takayasu Arteritis OR Takayasu’s arteritis) ) AND (upadacitinib))	65		0
		((Takayasu Arteritis OR Takayasu’s arteritis)) AND (tofacitinib)) AND ((effectiveness OR safety OR evidence OR efficacy OR use)	182		0
		((Takayasu Arteritis OR Takayasu’s arteritis)) AND (tofacitinib))	216		3
		((Takayasu Arteritis OR Takayasu’s arteritis) ) AND (ruxolitinib)) AND ((effectiveness OR safety OR evidence OR efficacy OR use)	53		0
		((Takayasu Arteritis OR Takayasu’s arteritis) ) AND (ruxolitinib))	53		0
		"JAK inhibitors" OR baricitinib OR upadacitinib OR tofacitinib OR ruxolitinib) AND "large vessel vasculitis" AND ((effectiveness OR safety OR evidence OR efficacy OR use)	256		0
		"JAK inhibitors" OR baricitinib OR upadacitinib OR tofacitinib OR ruxolitinib) AND "large vessel vasculitis"	264		1
Google Scholar	Takayasu arteritis, giant cell arteritis, JAK inhibitors, tofacitinib, upadacitinib, baricitinib, ruxolitinib, effectiveness, evidence, efficacy, safety, use	Janus kinase inhibitors “Takayasu arteritis”	1030	2019-2024 In the title of the articles with all the words	1
		Janus kinase inhibitors “Takayasu’s arteritis”	1010		0
		Tofacitinib “ Takayasu arteritis”	737		16
		tofacitinib "Takayasu's arteritis "	179		4
		Upadacitinib “Takayasu arteritis”	309		0
		Upadacitinib "Takayasu's arteritis "	40		0
		Baricitinib “Takayasu arteritis”	514		1
		Baricitinib "Takayasu's arteritis "	57		0
		Ruxolitinib “Takayasu arteritis”	169		0
		Ruxolitinib "Takayasu's arteritis "	18		0
		Janus kinase inhibitors “giant cell arteritis”	2340		5
		Tofacitinib “giant cell arteritis”	1210		0
		Upadacitinib “giant cell arteritis”	478		2
		Baricitinib “giant cell arteritis”	1080		3
		Ruxolitinib “giant cell arteritis”	402		1
Science Direct	Takayasu arteritis, giant cell arteritis, JAK inhibitors, tofacitinib, upadacitinib, baricitinib, ruxolitinib, effectiveness, evidence, efficacy, safety, use	"Takayasu arteritis" AND "JAK inhibitors" AND (effectiveness OR safety OR evidence OR efficacy OR use)	66	2019-2024 Research articles, Review articles, English	40
		"Takayasu arteritis" AND "tofacitinib" AND (effectiveness OR safety OR evidence OR efficacy OR use)	91		45
		"Takayasu arteritis" AND "upadacitinib" AND (effectiveness OR safety OR evidence OR efficacy OR use)	29		22
		"Takayasu arteritis" AND "baricitinib" AND (effectiveness OR safety OR evidence OR efficacy OR use)	56		37
		"Takayasu arteritis" AND "ruxolitinib" AND (effectiveness OR safety OR evidence OR efficacy OR use)	28		13
		"Giant Cell arteritis" AND “JAK inhibitors" AND (effectiveness OR safety OR evidence OR efficacy OR use)	196		116
		“Giant Cell Arteritis” AND "tofacitinib" AND (effectiveness OR safety OR evidence OR efficacy OR use)	215	2019-2024 Research articles, Review articles, Case reports, English	107
		"Giant Cell arteritis" AND “upadacitinib" AND (effectiveness OR safety OR evidence OR efficacy OR use)	95	2019-2024 Research articles, Review articles, English	60
		"Giant Cell arteritis" AND “baricitinib" AND (effectiveness OR safety OR evidence OR efficacy OR use)	164		94
		"Giant Cell arteritis" AND “ruxolitinib" AND (effectiveness OR safety OR evidence OR efficacy OR use)	91	2019-2024 Research articles, Review articles, English	48
Cochrane Library	Takayasu arteritis, giant cell arteritis, JAK inhibitors, tofacitinib, upadacitinib, baricitinib, ruxolitinib, effectiveness, evidence, efficacy, safety, use	"Takayasu arteritis" AND "JAK inhibitors" AND (effectiveness OR safety OR evidence OR efficacy OR use)	1	2019-2024	1
		"Takayasu arteritis" AND "tofacitinib"	6		6
		"Takayasu arteritis" AND "upadacitinib"	1		1
		"Takayasu arteritis" AND "baricitinib"	0		0
		"Takayasu arteritis" AND "ruxolitinib”	0		0
		“Giant cell arteritis" AND "JAK inhibitors"	1		1
		“Giant cell arteritis" AND "baricitinib"	4		4
		“Giant Cell Arteritis” AND "tofacitinib"	2		2
		"Giant Cell arteritis" AND “upadacitinib"	5		5
		"Giant Cell arteritis" AND “ruxolitinib"	0		0
Embase	Takayasu arteritis, giant cell arteritis, JAK inhibitors, tofacitinib, upadacitinib, baricitinib, ruxolitinib, effectiveness, evidence, efficacy, safety, use	1 Takayasu arteritis.mp. 2 Janus kinase inhibitor/ or baricitinib/ or ruxolitinib/ or tofacitinib/ or upadacitinib/ 3 comparative effectiveness/ or clinical effectiveness/ 4 drug efficacy/ 5 3 or 4 6 drug safety/ or safety/ 7 5 or 6 8 1 and 2 and 4 and 6	25	2019-2024	23
		1 Giant cell arteritis/dm, dt [Disease Management, Drug Therapy]. 2 janus kinase inhibitor/ or baricitinib/ or ruxolitinib/ or tofacitinib/ or upadacitinib/ 3 comparative effectiveness/ or clinical effectiveness/ 4 drug efficacy/ 5 3 or 4 6 drug safety/ or safety/ 7 5 or 6 8 1 and 2 and 4 and 6	34	2019-2024 Review Article	29
